# Fecal butyrate and deoxycholic acid quantitation for rapid assessment of the gut microbiome

**DOI:** 10.1371/journal.pone.0337727

**Published:** 2026-01-09

**Authors:** Michael W. Mullowney, Angelica Moran, Antonio Hernandez, Mary McMillin, Amber R. Rose, David Moran, Jessica Little, Ann B. Nguyen, Bhakti K. Patel, Christopher J. Lehmann, Matthew A. Odenwald, Eric G. Pamer, Kiang-Teck J. Yeo, Ashley M. Sidebottom

**Affiliations:** 1 Duchossois Family Institute, University of Chicago, Chicago, Illinois, United States of America; 2 Department of Pathology, University of Chicago, Chicago, Illinois, United States of America; 3 Department of Medicine, University of Chicago, Chicago, Illinois, United States of America; 4 Department of Microbiology, University of Chicago, Chicago, Illinois, United States of America; Laurentian University, CANADA

## Abstract

The intestinal microbiome is composed of myriad microbial species with impacts on host health that are mediated by the production of metabolites. While loss of bacterial species and beneficial metabolites from the fecal microbiome is associated with development of a range of diseases and medical complications, there are currently no clinical diagnostic tests that rapidly identify individuals with microbiome deficiencies. This method aims to rapidly quantify fecal concentrations of butyrate and deoxycholic acid, as depletion of these two metabolites are associated with adverse clinical outcomes and result from the loss of a subset of health-associated bacterial species. We present a rapid diagnostic screen based on 3-nitrophenylhydrazine derivatization and ultrahigh-performance liquid chromatography-mass spectrometry that measures fecal butyrate and deoxycholic acid concentrations as markers of microbiome function. A matrix-matched calibration curve was developed using a simulated fecal mixture to optimize accuracy and facilitate adherence to clinical laboratory regulations. The assay resulted in an analytical measurement range from 4.30–3030 µM (LLOQ = 3.71 µM) for butyrate and from 0.9–64.9 µM (LLOQ = 0.7 µM) for deoxycholic acid. Precision evaluation demonstrated a coefficient of variation <15% at all quality control levels tested. The rapid liquid chromatography-mass spectrometry screen can be performed in under an hour from extraction to provision of quantitative results, enabling the rapid identification of patients with defective microbiome function.

## Introduction

The intestinal microbiome is composed of trillions of microbes that include bacteria, viruses, fungi, and archaea that facilitate absorption of complex dietary components and provide resistance against infection by inhibiting pathogenic organisms, enhance epithelial barrier function, and optimizing immune defenses [1]. Commonly prescribed antibiotics can reduce the diversity of commensal microbial populations that constitute the microbiome. Furthermore, low-fiber diets limit microbiome functions, including production of health-promoting metabolites. Loss of microbiome diversity and microbially-produced metabolites has been associated with inflammatory bowel diseases such as Crohn’s disease and ulcerative colitis [2, 3], *Clostridioides difficile* infections [4], mortality following COVID-19 [5], graft-versus-host disease following hematopoietic stem cell transplantation [6] and systemic infections in patients with liver disease or following organ transplantation [7–9]. Clinical trials investigating the reconstitution of microbiome deficiencies are limited, in part because real-time identification of patients with compositional or functional microbiome deficiencies remains an unmet challenge. Two recent clinical trials aimed toward assessing the efficacy of a defined bacterial consortium, VE303, to modulate the gut microbiome and prevent recurrent *Clostridioides difficile* infection identified trial subjects based on broad pre-clinical parameters and cleared subjects’ existing microbiomes using vancomycin prior to treatment rather than measuring and treating subjects based on their existing, real-time microbiota function or composition [10, 11]. Fecal nucleic acid sequencing and metagenomic analyses can determine microbiome compositions and mass spectrometry can quantify fecal metabolites, but these platforms have yet to be developed into clinical tests that rapidly identify patients with deficient microbiome compositions or functions [12].

**Fig 1:**
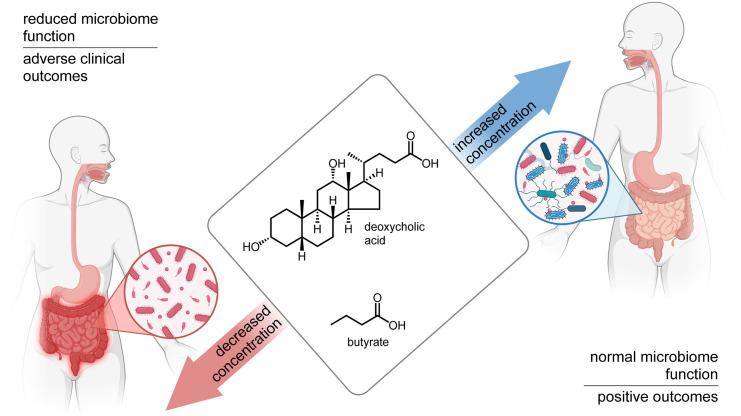
Reduced fecal butyrate and deoxycholic acid (DCA) in hospitalized patients are associated with adverse medical outcomes. A same-day fecal screen would enable identification of patients with the low butyrate and DCA levels indicative of higher risk for adverse events in a clinically relevant timeframe.

While the intestinal microbiome produces thousands of distinct metabolites, short-chain fatty acids (SCFAs) and bile acids are particularly impactful on host physiology and immune defenses. Butyrate, a SCFA, is produced by obligate anaerobic bacterial species in the lower gastrointestinal tract and impacts immune system development [1]. Primary bile acids are produced and conjugated to taurine and glycine in the liver and facilitate nutrient absorption upon delivery to the small intestine. Intestinal microbes then modify primary bile acids through deconjugation and hydroxylation to secondary bile acids. Secondary bile acids, such as deoxycholic acid (DCA) and its microbially-generated derivatives, impact host physiology and immune cell differentiation [1]. Median concentrations of 12.53 mM (IQR 9.395–21.19 mM) and 940.16 µM (IQR 371.78–1814.76 µM) for butyrate and DCA, respectively, have been reported in fecal samples from a healthy donor cohort [13]. Recent studies by our group and others have demonstrated that fecal butyrate and DCA concentrations are commonly reduced in hospitalized patients (liver disease, COVID-19, liver transplant, and heart transplant) and are associated with a range of adverse medical outcomes (Fig 1) [1, 4–9, 13]. Because lower fecal butyrate and DCA levels are associated with adverse clinical outcomes, the development of a rapid laboratory test to identify patients with microbiome deficiencies may, in the near-term, facilitate clinical studies of microbiome-targeting treatments and, in the long-term, identify vulnerable patients for microbiome-enhancing therapies.

**Fig 2:**
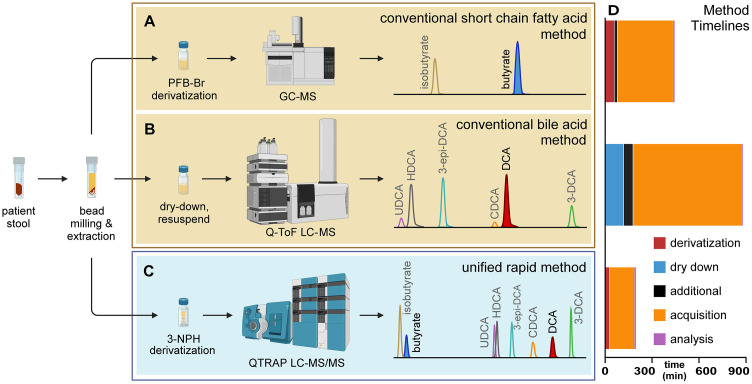
Conventional compared to rapid measurement of butyrate and DCA. Comparison of conventional, targeted short-chain fatty acid and bile acid metabolomics methods, such as those in academic labs **(A, B)**, to the unified measurement of these classes on one mass spectrometry platform **(C)**. Conventional methods require multiple mass spectrometry platforms to measure metabolites with dissimilar chemical characteristics. **(A)** Short-chain fatty acids (SCFAs) such as butyrate typically require analysis by GC-MS, often with derivatization, while (B) bile acids are routinely analyzed using LC-MS technology. **(C)** The rapid mass spectrometry method presented here unifies the measurement of SCFAs and bile acids while resolving multiple isomers on a single system that minimizes sample preparation, cost (instrumentation, personnel time) and data acquisition time. **(D)** Sample preparation, data acquisition, and analysis time to acquire quantitative butyrate and deoxycholic acid concentration data for one biological sample using conventional metabolomics methods and the rapid metabolomics screen (See also S2 Table in S1 File).

Given the temporally dynamic nature of the intestinal microbiome compositions impacted by events such as antibiotic exposure or disease onset, practical applications and treatments based on an individual’s real-time microbiome function will require rapid quantitation of fecal metabolite concentrations. SCFAs are commonly measured by gas chromatography-mass spectrometry (GC-MS) following derivatization with pentafluorobenzyl bromide (PFB-Br) or trimethylsilyl (TMS)-donating reagents such as *N*,*O*-bis(trimethylsilyl)trifluoroacetamide (BSTFA; Fig 2A) [14, 15]. Bile acids are commonly separated and analyzed with liquid chromatography-MS (LC-MS) systems using sample preparation that may include steps to optimize signal such as dry down and resuspension, but without derivatization (Fig 2B) [16]. LC-MS-based platforms allow sample preparation and analysis in significantly less time compared to GC-MS methods, but SCFAs suffer from poor recovery, stability, and ionization in conventional LC-MS methods [17]. Conversely, larger metabolites such as bile acids lack the volatility for efficient and sensitive GC-based analysis. Among the requirements for a method encompassing both classes of metabolites were the applicability to one LC-MS-based system with one injection, a single calibration curve, a single set of matched internal standards, and quality control samples that accounted for all chemical species. Additionally, a single test decreases sample preparation time, instrument time, instrument cost, personnel time, and analysis time. The common chemical characteristic between the SCFAs and bile acids is the carboxylic acid functional group. With this in mind, we identified and halved the run time of a method involving derivatization of the carboxylic acid moiety by 3-nitrophenylhydrazine (3-NPH) catalyzed by *N*-(3-dimethylaminopropyl)-*N*′-ethylcarbodiimide hydrochloride (EDC) [18], enabling rapid measurement of both SCFAs and bile acids, specifically butyrate and deoxycholic acid, on the same LC-MS/MS platform (Fig 2C, D).

There is a growing need to develop rapid screening or diagnostic tools to identify patients with decreased microbiome function. There are no commercial tests cleared by the US Food and Drug Administration (FDA) for assessing the gut microbiome, whether for specific microbes and microbiome-derived metabolites, despite an increasing number of clinical trials seeking to modify, diversify or optimize the microbiome. Accurately characterizing microbiome abnormalities will be critical for the design and success of these trials, given the complexity and variability of the intestinal microbiome, with specific compositional changes resulting in specific clinical ramifications.

The test is classified as a custom-designed laboratory developed test (LDT) and can be validated to meet the requirements of the Clinical Laboratory Improvement Amendments (CLIA) of 1988 and College of American Pathologists (CAP). LDTs are not required to be FDA-cleared for research purposes, however, they are subject to federal regulations such as CLIA if a laboratory wishes to use it for any testing that will direct patient care [19]. To adhere to CAP requirements for validation, we developed a matrix-matched material or simulated fecal matrix for validation of the LDT method [20]. The matrix-matched materials are used in preparation of the calibration curve and quality control samples to more accurately quantify the endogenous compounds of interest. There is no commercially available fecal material matrix that lacks butyrate and DCA. We chose to generate a simulated fecal matrix to spike in known concentrations of butyrate and DCA to create this necessary sample type. While simulated fecal matrices are being developed for metagenomic sequencing, matrices specific for targeted metabolomics are currently unavailable; therefore, we have created a simulated matrix from commonly available food products that lack our target analytes but compositionally reflect fecal material. In this study, we have optimized and clinically validated a LC-MS/MS method to quantify butyrate and DCA for assessment of gut microbiome health. With the ability for same-day identification of patients with reduced microbiome function, this method can be used to select and monitor patients in clinical research trials.

## Materials and methods

### Materials

A solution of 80% LC-MS grade methanol (Fisher Scientific; Optima A456) with 500 µM sodium D_7_-butyrate (98%, Cambridge Isotope Laboratories; DLM-7616) and 2.55 µM D_4_-deoxycholic acid (98%, Cambridge Isotope Laboratories; DLM-2824) as internal standards (ISTDs) was prepared for extraction of all samples.

LC-MS/MS methods were optimized and calibration curves were built using authenticated standards of sodium acetate (Sigma Aldrich; S2889), sodium propionate (Sigma Aldrich; P1880), sodium butyrate (Sigma Aldrich; B5887), isobutyric acid (Sigma Aldrich; I1754), valeric acid (Sigma Aldrich; 240370), 3-(4-hydroxyphenyl)propionic acid (desaminotyrosine; DAT; Sigma Aldrich; H52406), indole-3-carboxaldehyde (I3C; Sigma Aldrich; 129445), cholic acid (Sigma Aldrich; C1129), glycocholic acid (GCA; Sigma Aldrich; G2878), deoxycholic acid (DCA; Sigma Aldrich; D2510), ursodeoxycholic acid (UDCA; Sigma Aldrich; U5127), hyodeoxycholic acid (HDCA; Sigma Aldrich; H3878), 5β-cholanic acid-3β,12α-diol (3-epi-deoxycholic acid; 3-epi-DCA; Steraloids, Inc.; C1165-000), chenodeoxycholic acid (CDCA; Sigma Aldrich; C9377), 5β-cholanic acid-7α,12α-diol (3-deoxycholic acid; 3-DCA; Steraloids, Inc.; C1170-000), taurocholic acid (TCA; Sigma Aldrich; T4009), tauro-α-muricholic acid (Avanti Polar Lipids; 700243P), sodium 5β-cholanic acid-3α,6β,7β-triol *N*-(2-sulphoethyl)-amide (tauro-β-muricholic acid; Steraloids, Inc.; C1899-000), alloisolithocholic acid (Avanti Polar Lipids; 700196), isolithocholic acid (isoLCA; Avanti Polar Lipids; 700195P), and lithocholic acid (LCA; Sigma Aldrich; L6250).

Derivatization reagents were *N*-(3-dimethylaminopropyl)-*N*′-ethylcarbodiimide hydrochloride (EDC; Sigma-Aldrich; E7750) and 3-nitrophenylhydrazine hydrochloride (3-NPH; Sigma-Aldrich; N21804). Solvents used were LC-MS grade water (Fisher Scientific; Optima W7), LC-MS grade acetonitrile (Fisher Scientific; Optima A955), LC-MS grade 2-propanol (Fisher Scientific; Optima A461), and 99% Ultra-Pure LC-MS Grade formic acid (CovaChem; 11202) as a mobile phase buffer.

The simulated fecal matrix was comprised of non-organic avocado (Trader Joe’s), Gluten-Free All Purpose Flour (comprised of millet flour, potato starch, tapioca starch, rice flour, sorghum flour, and xanthan gum; Trader Joe’s), canned 365 Whole Foods Market organic black beans (Whole Foods Market), Clabber Girl Cornstarch (Jewel-Osco Grocery), and Xclusiv Organics cosmetic grade snail mucilage extract powder (Walmart.com, online).

### Simulated fecal matrix

Two microbiota-derived metabolites, butyrate and DCA, were quantified in food products and snail mucin using LC-MS/MS for development of a CAP required matrix-matched sample. Food items with endogenous concentrations of these metabolites below the limit of quantitation were selected to create a simulated fecal matrix with a consistency similar to human stool (data not shown). The simulated fecal mixture consisted of equal parts by weight of avocado, gluten-free all-purpose flour, black beans, and cornstarch, to which the snail mucilage powder was added at 0.5% (w/w) concentration. The solid components were blended until smooth in a conventional household blender and diluted with 0.7 mL deionized Milli-Q water per gram of solid mixture. These raw food ingredients were selected to approximate the ratio of carbohydrates, proteins, fats, and fiber found in human stool [21]. Though it is known that human feces contains mucin [22], to our knowledge there is no report of a normal fecal mucin concentration range in adults. Snail mucin was selected to simulate human fecal mucin over other animal-based mucins due to its lack of DCA or butyrate. The optimal simulated fecal matrix snail mucin concentration of 0.5% (w/w) was determined by comparing the recovery of spiked DCA and butyrate from three fecal samples without endogenous DCA and butyrate versus our simulated matrix containing a range of snail mucin concentrations (data not shown).

### Sample extraction and preparation

Following collection, all stool samples were stored at −80 °C until further processed. Extraction solvent (80% methanol spiked with internal standards purchased from Cambridge Isotope Laboratories) was added to all pre-weighed samples to 100 mg/mL in bead beating tubes (Fisherbrand Bead Ruptor; 15-340-154). Samples were homogenized at 4 °C on a Bead Mill 24 Homogenizer (Fisher; 15-340-163), set at 1.6 m/s with six 30-second cycles, 5 seconds off per cycle. Samples were then centrifuged at −10 °C, 20,000 x *g* for 15 minutes and the supernatant was used for subsequent metabolomic analysis.

### Conventional metabolite analysis using GC-MS with PFB-Br derivatization

SCFAs were derivatized as described by Haak, *et al.* with the following modifications [14]. Metabolite extract (100 µL) was added to borate buffer (100 µL) (pH 10) (Thermo Fisher, 28341), 400 µL of 100 mM pentafluorobenzyl bromide (Millipore Sigma; 90257) in acetonitrile, and 400 µL of *n*-hexane (Acros Organics; 160780010) in a capped MS autosampler vial (Microliter; 09–1200). Samples were heated in a thermomixer C (Eppendorf) to 65 °C for 1 hour while shaking at 1300 rpm. After cooling to room temperature, samples were centrifuged at 4 °C, 2000 x *g* for 5 minutes, allowing phase separation. From the hexanes phase (top layer), 100 µL was transferred to an autosampler vial containing a glass insert and the vial was sealed. Another 100 µL of the hexanes phase was diluted with 900 µL of *n*-hexane in an autosampler vial. Concentrated and dilute samples were analyzed using a GC-MS (Agilent 7890A GC system, Agilent 5975C MS detector) operating in negative chemical ionization mode, using a HP-5MSUI column (30 m x 0.25 mm, 0.25 µm; Agilent Technologies 19091S-433UI), methane as the reagent gas (99.999% pure) and 1 µL split injection (1:10 split ratio). Oven ramp parameters: 1 min hold at 60 °C, 25 °C per min up to 300 °C with a 2.5 min hold at 300 °C. Inlet temperature was 280 °C and transfer line was 310 °C. A 10-point calibration curve was prepared starting from 12.5 mM sodium butyrate in water with nine subsequent 2x serial dilutions. Data analysis was performed using MassHunter Quantitative Analysis software (version B.10, Agilent Technologies) and confirmed by comparison to authentic standards. Normalized peak areas were calculated by dividing raw peak areas of targeted analytes by averaged raw peak areas of internal standards.

### Conventional bile acid analysis using QToF LC-MS

Bile acids were analyzed using LC-MS. A 75 µL aliquot of metabolite extract was added to an MS autosampler vial (Microliter; 09–1200) and dried down completely under a nitrogen stream at 30 L/min (top) 1 L/min (bottom) at 30 °C (Biotage SPE Dry 96 Dual; 3579M). Samples were resuspended in 750 µL of 50:50 water:methanol. Vials were added to a thermomixer C (Eppendorf) to resuspend analytes at 4 °C, 1000 rpm for 15 min with an infinite hold at 4 °C. Samples were transferred to microcentrifuge tubes and centrifuged at 4 °C, 20,000 x *g* for 15 min to remove insoluble debris. A 700 µL portion of supernatant was transferred to a fresh, pre-labeled MS vial. Samples were analyzed on an Agilent 1290 infinity II liquid chromatography system coupled to an Agilent 6546 quadrupole time-of-flight (QToF) mass spectrometer operating in negative mode, equipped with an Agilent Jet Stream Electrospray Ionization source.

A sample volume of 5 µL was injected onto an Xbridge© BEH C18 Column (3.5 µm, 2.1 x 100 mm; Waters Corporation, 186003022) fitted with an XBridge© BEH C18 guard (Waters Corporation, 186007766) at 45 °C. Mobile phase A was water with 0.1% formic acid and mobile phase B was acetone with 0.1% formic acid. Gradient elution started with 28% B with a flow rate of 0.4 mL/min for 1 min and linearly increased to 33% B over 5 min, then linearly increased to 65% B over 14 min. Then the flow rate was increased to 0.6 mL/min and B was increased to 98% over 0.5 min. These conditions were held constant for 3.5 min. Re-equilibration was completed at a flow rate of 0.4 mL/min of 28% B for 3 min. The electrospray ionization conditions were set with the capillary voltage at 3.5 kV, nozzle voltage at 2 kV, and detection window set to mass-to-charge ratio (*m/z*) 100–1700 with continuous infusion of a reference mass (Agilent ESI TOF Biopolymer Analysis Reference Mix) for mass calibration.

A ten-point calibration curve was prepared with 318.4 µM deoxycholic acid in water, with nine subsequent 3x serial dilutions. Data analysis was performed using MassHunter Profinder Analysis software (version B.10, Agilent Technologies) and confirmed by comparison with authentic standards. Normalized peak areas were calculated by dividing raw peak areas of targeted analytes by averaged raw peak areas of internal standards.

### Rapid metabolomic screen using LC-MS

**Fig 3:**
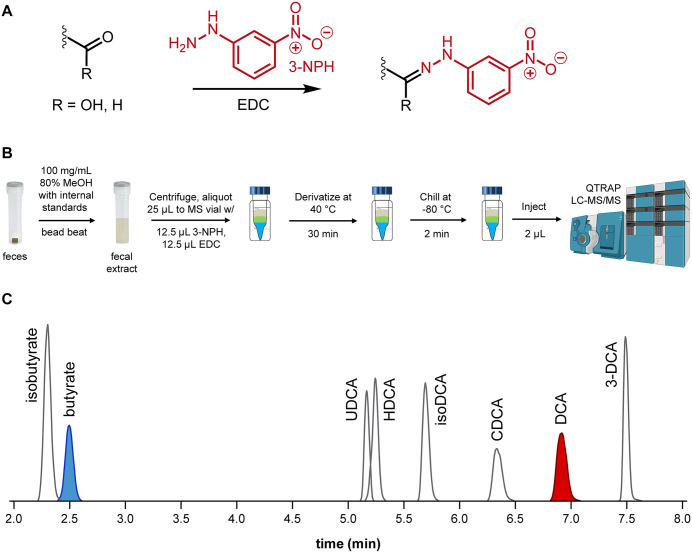
3-NPH derivatization mechanism. **(A)** Reaction mechanism of carboxylic acid and aldehyde derivatization with 3-nitrophenylhydrazine (3-NPH) catalyzed by *N*-(3-(dimethylamino)propyl)-*N*′-ethylcarbodiimide (EDC). **(B)** A five-step procedure to derivatize and prepare patient fecal samples for LC-MS/MS analysis. **(C)** A 10-minute chromatographic method enables the detection and resolution of butyrate and deoxycholic acid (DCA) from their isomers in the same sample injection.

Based on methods from Liao, *et al* [18], 25 µL of samples were transferred to a mass spectrometry vial with 12.5 µL 3-nitrophenylhydrazine (3-NPH) and 12.5 µL N-(3-dimethylaminopropyl)-N′-ethylcarbodiimide hydrochloride (EDC; Fig 3A). Samples were incubated at 40 °C for 30 minutes and then chilled at −80 °C for 2 minutes to complete the derivatization process. For optimal separation of isomers, 2 µL of sample was injected and separated on a CORTECS T3 column (Waters, 120Å, 1.6 µm, 2.1 x 100 mm) fitted with a CORTECS T3 VanGuard Pre-column (Waters, 120Å, 1.6 µm, 2.1 mm X 5 mm) at 45°C with a flow rate of 0.550 mL/min and run-time of 10 min using a multiple reaction monitoring (MRM)-based targeted analysis on a Sciex Exion LC AD system (Fig 3B). Mobile phase A was water with 0.1% formic acid and mobile phase B was isopropanol: acetonitrile (3:1) with 0.1% formic acid. Separation began with a 1 min hold at 15% B which was followed by 15–48% B from 1–4 min, 48% B for 2 min, 48–100% B from 6–8 min, 100% B from 8–9 min, 100–15% B from 9–9.1 min, and 15% B from 9.1–10 min. Analytes were detected by negative ionization using a Sciex QTRAP 6500 mass spectrometer with an electrospray ionization source. The method parameters were as follows, declustering potential: −60 V, entrance potential: −10 V, collision cell exit potential: −13 V, curtain gas: 30 psi, collision gas: 12 psi, ion spray voltage: −4500 V, temperature: 325 °C, ion source gas 1: 40 psi, and ion source gas 2: 40 psi.

Data analysis was performed using MultiQuant^TM^ software (version 3.0, Sciex). The precursor, quantifier product, and qualifier ions and retention times (± 0.2 min) for 3-NPH derivatized metabolites of interest were 3-NPH-butyrate (precursor: *m/z* 222.1, quantifier: *m/z* 137.1, qualifier: *m/z* 152.0, 2.40 min) and 3-NPH-DCA (precursor: *m/z* 526.3, quantifier: *m/z* 152.0, qualifier: *m/z* 137.1, 6.81 min). Derivatized biologically relevant isomers were measured based on detection of the same ions with unique retention times when compared to butyrate (isomer: isobutyrate) and deoxycholic acid (isomers: UDCA, HDCA, 3-DCA, CDCA, and DCA; Fig 3C). Acquisition parameters can be found in S1 Table in S1 File. For quantification, peak areas of 3-NPH derivatized metabolites in each sample were normalized to the peak areas of their respective internal standards (D_7_-3-NPH-butyrate for butyrate, D_4_-3-NPH-DCA for DCA).

### Preparation of calibrators and quality control (QC) materials for the rapid metabolomic screen

Calibrators and QCs were created by spiking butyrate and DCA into either 50% methanol (for conventional metabolite analyses) or simulated fecal matrix (rapid metabolomic screen validation, this study). A set of seven calibrators were prepared for butyrate (50–4000 µM) and DCA (1–80 µM). QCs were independently prepared to five concentration levels of butyrate (125, 375, 750, 1250, and 2500 µM) and DCA (2.5, 7.5, 15, 25, 50 µM).

### Rapid metabolomic screen analytical measurement range (AMR) and lower limit of quantification (LLOQ)

The analytical measurement range (AMR) was determined by creating a set of simulated fecal matrix samples with butyrate and DCA at concentrations ranging from 2.25–5000 µM and 0.15–1.5 µM, respectively. The measured concentrations were compared to the expected concentrations to assess linearity. The lower limit of quantitation (LLOQ) was determined by running a set of 6 low concentration simulated samples over 3 days (8 replicates/day) and defined as concentration at which the % CV across replicates is < 20%.

### Rapid metabolomic screen precision and recovery

Intra-day and inter-day precision was assessed using QC materials with known amounts of butyrate and DCA in the simulated fecal matrix. QCs were measured 10 times in a single run to determine intra-day precision. Inter-day reproducibility was determined by testing QCs in duplicate daily for a total of 14 replicates. CV of <15% at each level was considered acceptable. Accuracy was assessed by calculating the recovery of each analyte relative to the expected concentrations.

### Method comparison

Clinical samples were tested using conventional metabolite analysis by PFB-Br derivatization with GC-MS, conventional bile acid analysis by LC-MS, and the rapid metabolomic screen. Results from samples within the analytical measurement range of all methods were analyzed using linear regression and Bland–Altman bias plots to determine the agreement between methods.

### Clinical studies

Approval by the University of Chicago Institutional Review Board was granted for the clinical studies that were the source of the samples analyzed herein (liver disease, IRB21–0327; liver transplant, IRB 20–0163; heart transplant, IRB20–0333; medical intensive care unit, IRB20–1102). The recruitment period for each study is as follows: 1.) Liver disease, IRB21–0327: 4/6/2021 – Ongoing, 2.) Liver transplant, IRB 20–0163: 6/22/2020–4/25/2023, 3.) Heart transplant, IRB20–0333: 7/1/2020–11/5/2024, 4.) Medical intensive care unit, IRB20–1102: 9/19/2020–5/21/2021. Written informed consent was obtained for all studies. These studies do not include minors. These studies are not retrospective. All data was captured prospectively, and authors did not have access to information that can identify individual patients.

## Results

**Fig 4:**
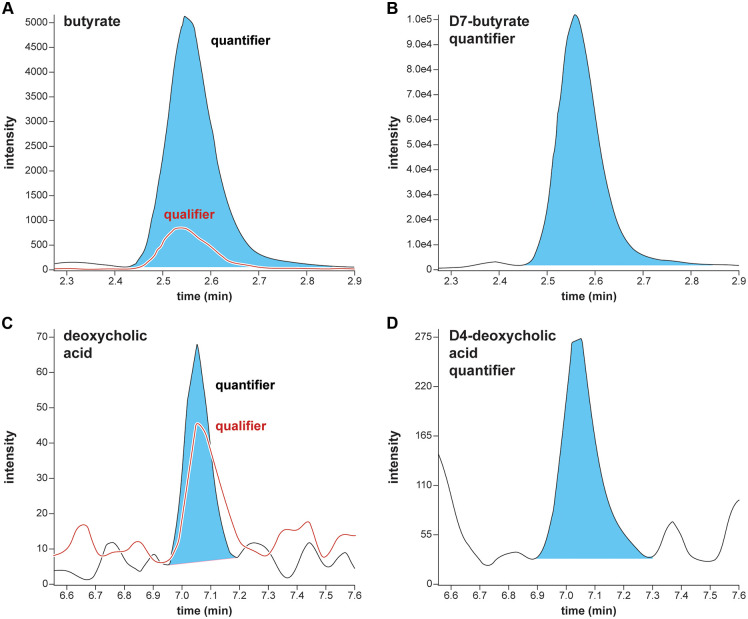
MRM butyrate and DCA chromatograms. Multiple reaction monitoring chromatogram overlays from the lowest rapid metabolomic screen calibrator for butyrate (A; quantifier, *m/z* 222.1–137.1; qualifier, *m/z* 222.1–152.0) with D_7_-butyrate internal standard quantifier transition (B; *m/z* 229.1–137.1) and deoxycholic acid (C; quantifier, *m/z* 526.3–152.0; qualifier, *m/z* 526.3–137.1) with D_4_-deoxycholic acid internal standard quantifier transition (D; *m/z* 530.3–137.1).

Butyrate concentrations in experimental samples were determined using the butyrate quantifier transition of *m/z* 222.1 to 137.1 normalized to the D_7_-butyrate internal standard quantifier mass transition of *m/z* 229.1 to 137.1. Chromatogram overlays from the second lowest calibrator (50 µM) in the matched matrix for butyrate quantifier and qualifier transitions with the D_7_-butyrate internal standard (500 µM) quantifier transition are shown in Fig 4A and B. DCA concentrations in experimental samples were determined using the DCA quantifier transition of *m/z* 526.3 to 152.0 normalized to the D_4_-DCA internal standard quantifier mass transition of *m/z* 530.3 to 137.1. The product ions for quantifier and qualifier transitions were always either *m/z* 152.0 or 137.1 as they arise from a fragment of 3-NPH and were the most abundant. Chromatogram overlays from the lowest calibrator (1 µM) in matched matrix for both deoxycholic acid quantifier and qualifier transitions with the D_4_-DCA internal standard (2.5 µM) quantifier transition are shown in Fig 4C and D.

### AMR and LLOQ

**Fig 5:**
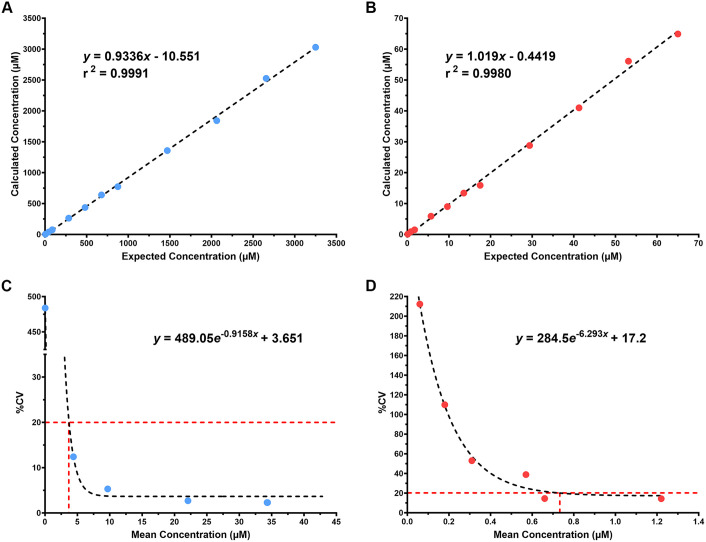
Validation metrics for quantifying butyrate and DCA from fecal sample. The analytical measurement ranges (AMR) and linearity of butyrate (A) and DCA (B) are shown across eleven concentration points for the rapid metabolomic screen. The lower limit of quantification (LLOQ) for butyrate at 3.71 µM (C) and DCA at 0.7 µM (D) are defined by the concentration at which the percent coefficient of variation (% CV) reaches 20%.

The rapid metabolomic screen demonstrates an AMR from a butyrate concentration of 4.30–3030 µM with an r^2^ value of 0.9991 and from a DCA concentration of 0.9–64.9 µM with an r^2^ value of 0.9980 (Fig 5A, B). Using a % CV cutoff of 20%, the lower limit of quantification (LLOQ) was determined to be 3.71 µM for butyrate and 0.7 µM for deoxycholic acid (Fig 5C, D).

### Precision and accuracy

The reproducibility of this assay is excellent, with an inter-day precision of <15% CV at all five levels tested across the analytical measurement range (Table 1). Recovery ranged from 98.2–109% for butyrate and 92.4–109.7% for DCA, demonstrating accuracy of analyte measurement relative to expected concentrations using simulated fecal samples. Additional performance measures of the rapid metabolomic screen can be found in S3 Table in S1 File.

**Table 1:** Inter-day precision and accuracy for five concentration levels (L1–L5) spanning the analytical measurement range of the rapid metabolomic screen. Concentrations are shown in μM; % CV = percent coefficient of variation.

Butyrate	L1	L2	L3	L4	L5
Mean Concentration	123	384	758	1365	2596
Standard Deviation	12.8	14.5	42.4	33.1	121
% CV	0.104	0.0377	0.0559	0.0243	0.0465
**Expected Concentration**	125	375	750	1250	2500
**Recovery**	98.2%	102%	101%	109%	104%
**DCA**	**L1**	**L2**	**L3**	**L4**	**L5**
Mean Concentration	2.7	7.4	14.0	25.9	46.2
Standard Deviation	0.4	0.7	1.0	2.5	4.9
%CV	0.1	0.1	0.1	0.1	0.1
**Expected Concentration**	2.5	7.5	15	25	50
**Recovery**	109.7%	99.2%	93.3%	103.6%	92.4%

### Method comparison

**Fig 6:**
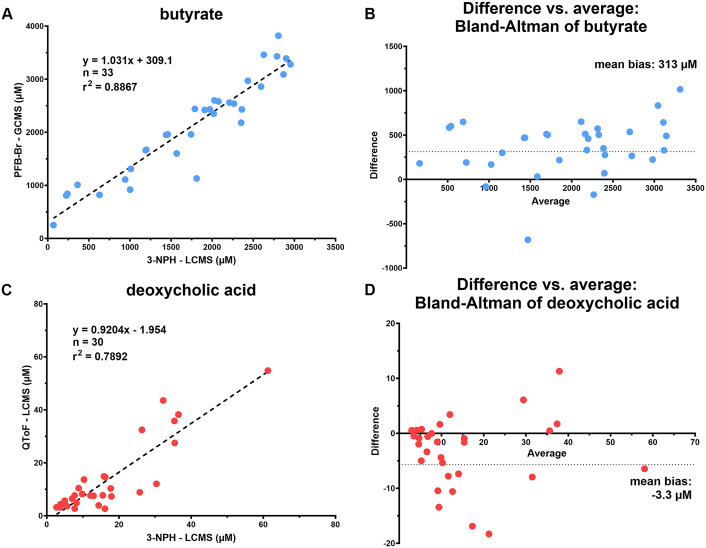
Comparison of the rapid metabolomic screen versus measurement by conventional mass spectrometry methods in patient fecal samples. Linear regression (A) and Bland-Altman (B) comparisons of butyrate concentrations as measured by the rapid metabolomic screen versus measurement by conventional GC-MS metabolite analysis with PFB-Br derivatization (GCMS); Linear regression (C) and Bland-Altman (D) comparisons of DCA concentrations as measured by the rapid metabolomic screen versus measurement by conventional bile acid analysis using QToF LC-MS. See S4 Table in S1 File for patient butyrate and DCA concentrations with clinical metadata.

A total 78 patient fecal samples from patients enrolled in microbiome studies of MICU, heart transplant, liver disease, and liver transplant populations were used for method comparison and rapid screen validation. Fecal samples were tested using our conventional metabolite analysis by GC-MS with PFB-Br derivatization, our conventional bile acid analysis by LC-MS, and the newly optimized rapid metabolomic screen. Within this set, there were 33 patients with butyrate concentrations within the AMR of both the rapid metabolomic screen and our conventional GC-MS with PFB-Br method, while there were 30 patients with DCA concentrations within the AMR of both the rapid metabolomic screen and the conventional QToF LC-MS-based method. For each patient, the butyrate concentration measured by the rapid metabolomic screen and conventional GC-MS analysis were compared for linearity and bias (Fig 6A, B) and showed an overall bias of 313 µM (Bland-Altman analysis). A comparison of the DCA concentrations measured using the rapid metabolomic screen and the conventional bile acid LC-MS analysis to assess linearity and bias showed a bias of −3.3 µM (Fig 6C, D).

## Discussion

We have developed a LDT for rapid metabolomic screen to identify patients with metabolically abnormal fecal microbiota. Fecal microbiome metabolomics has promise in evaluating patient health, but current methods are time-intensive and not widely implemented outside of research settings. The chemical characteristics of different target metabolites often require specific and differing sample preparation and instrumentation platforms for optimal, efficient, and reproducible quantitation. Thus, measuring multiple metabolites in a single screen is problematic because the optimal method for one metabolite is often incompatible with another. The method we developed herein accelerates the quantitation of two key fecal metabolites associated with clinical outcomes, butyrate and DCA, by optimizing previously reported 3-NPH derivatization chemistry and MRM-based QTRAP quantitation and establishes an LC-MS/MS platform that provides same-day insights into the fecal microbiome’s function. Our new screen yields patient butyrate and DCA quantitation results in less than a quarter of the total analysis time required using our conventional methods (210 min vs 867 min; Fig 3; S2 Table in S1 File) and in half the chromatographic time as existing 3-NPH-based platforms (10 min vs. 20 min) [18].

While the metabolomic screen we describe will rapidly identify patients with dysfunctional microbiomes for enrollment in clinical trials of microbiome reconstitution, it also represents a step in the direction of personalizing microbiome-targeting interventions by targeting specific defects. We demonstrate that additional microbiome-relevant metabolites beyond butyrate and DCA can be quantified by the rapid screen (S1 Fig in S1 File), enabling assessment of more complex patterns of microbiome health and facilitating a more personalized approach. In addition to expanding the set of metabolites measured in fecal samples, the test can be adapted for other biological sample types, such as plasma or urine, which are more readily acquired from patients.

While butyrate and DCA are biologically relevant and directly measuring them is useful regardless of the underlying cause, the rapid metabolomic screen is unable to distinguish between patients with low concentrations of butyrate attributable to depleted microbiome diversity versus low prebiotic fiber intake or other causes unrelated to microbiome composition. When the goal is to rapidly assess for microbial composition disturbances, the addition of quantitative polymerase chain reaction (qPCR) targeted for specific organisms may be a useful complement to the rapid metabolomic method.

We observed differences in the quantitation of butyrate and DCA between the rapid screen and our conventional platforms. There is a positive bias when comparing 3-NPH derivatization and MRM-based QTRAP measurement of butyrate in the rapid metabolomic screen to our conventional measurements using PFB-Br derivatization and GC-MS. While differing reaction efficiencies between 3-NPH and PFB-Br may contribute to these variations, bias could be impacted due to sensitivity differences between the instrument mass analyzers. The MRM-based targeted QTRAP analysis in the rapid metabolomic screen allows for much higher sensitivity in the detection of butyrate, with an LLOQ at 3.71 µM, compared to an LLOQ of 750 µM in the conventional GC-MS platform. Fecal samples with lower butyrate concentrations are measured more accurately on the rapid metabolomic screen resulting in a bias due to increased sensitivity compared to the GC-MS. A negative bias is observed when comparing the MRM-based QTRAP mass spectrometer measurement of DCA in the rapid metabolomic screen to our conventional measurements using a QToF. The rapid metabolomic screen employs the 3-NPH derivatization reagent with highly selective MRM-based QTRAP detection of precursor to product ion transitions, which results in a very high signal-to-noise ratio. In contrast, the QToF analyzer is scanning for ions at high resolution across a wide *m/*z range (100–1700) which decreases signal-to-noise. These biases are negligible compared to typical concentrations of butyrate and DCA observed in healthy donor populations [13] but may complicate comparisons across research studies on diseased populations where different methodologies are used.

The development of a rapid, sensitive and robust LC-MS/MS based screen for microbiota-derived metabolites represents a major improvement in the clinical assessment of the microbiome in patients that can be validated for a CLIA/CAP regulated environment. Although advances in genomics allow comprehensive investigation of the gut microbiome, sample preparation and analysis are lengthy and require significant laboratory and bioinformatic resources unavailable to most hospitals. With the increased use of LC-MS/MS technology in clinical chemistry laboratories, easily accessible reagents, and minimal sample preparation procedures, the rapid metabolic screen described herein can be readily implemented as a clinical test to laboratories licensed for patient testing.

In summary, the unified LC-MS/MS-based metabolomic screen we have developed rapidly quantifies butyrate and DCA from fecal samples to measure gut microbiome functional dysbiosis. This screen has the flexibility to measure additional microbiome-relevant metabolites with carboxylic acids or aldehydes, such as SCFA and bile acids beyond butyrate and DCA, which may be relevant to patients with specific diseases (S1 Fig in S1 File). The simulated fecal matrix presented here serves as an analyte-free surrogate for patient stool, enabling development, validation, and adherence to regulatory and accreditation standards for clinical assays to assess health of the gut microbiome.

### Approval for clinical sample collection

Approval by the University of Chicago Institutional Review Board was granted for the clinical studies that were the source of the samples analyzed herein (liver disease, IRB21–0327; liver transplant, IRB 20–0163; heart transplant, IRB20–0333; medical intensive care unit, IRB20–1102).

## Supporting information

S1 File Additional information on mass spectrometry details, method comparison between conventional and rapid screen for butyrate and deoxycholic acid quantitation, and overview of additional microbiome relevant metabolites that can be measured using the rapid screen. (PDF)
