# Supplementary material for: Fecal butyrate and deoxycholic acid quantitation for rapid assessment of the gut microbiome
Source: PLoS One. 2026 Jan 9;21(1):e0337727. doi: 10.1371/journal.pone.0337727 (PMC12788677; doi:10.1371/journal.pone.0337727)
Supplement: S1 File — (PDF) [file pone.0337727.s001.pdf]

Supplemental Information

Fecal butyrate and deoxycholic acid quantitation for rapid assessment of the gut microbiome

Michael W. Mullowney[1], Angelica Moran[1,2], Antonio Hernandez[1], Mary McMillin[1], Amber R. Rose[1], David Moran[1], Jessica Little[1], Ann B. Nguyen[3], Bhakti K. Patel[3], Christopher J. Lehmann[1,3], Matthew A. Odenwald[1,3], Eric G. Pamer[1,3,4], Kiang-Teck J. Yeo[2], Ashley M. Sidebottom[1*]

[1]Duchossois Family Institute, University of Chicago, Chicago, Illinois, United States of America

[2]Department of Pathology, University of Chicago, Chicago, Illinois, United States of America

[3]Department of Medicine, University of Chicago, Chicago, Illinois, United States of America

[4]Department of Microbiology, University of Chicago, Chicago, Illinois, United States of America

*Corresponding author email: asidebottom@bsd.uchicago.edu

*Corresponding author postal address: 900 E 57th St., KCBD 4130, Chicago, IL 60637, USA

Table S1. Data acquisition parameters for the Sciex QTRAP used in the rapid metabolomic screen.

Metabolite	Precursor ion (Q1 *m/z*; Da)	Quantifier product ion (Q3 *m/z*; Da)	Qualifier product ion (Q3 *m/z*; Da)	Retention time (min)	Acquisition window (sec)	Dwell time weight	Collision energy (eV)
3NPH-isobutyrate	222.09	137.1	152.0	2.2	30	1	-30
3NPH-butyrate	222.09	137.1	152.0	2.5	30	1	-30
3NPH-UDCA[a]	526.33	152.0	137.1	5.1	30	1	-65
3NPH-HDCA[b]	526.33	152.0	137.1	5.2	30	1	-65
NPH-isoDCA[c]	526.33	152.0	137.1	5.6	30	1	-65
NPH-CDCA[d]	526.33	152.0	137.1	6.2	30	1	-65
NPH-DCA[e]	526.33	152.0	137.1	6.8	30	1	-65
3NPH-3-DCA[f]	526.33	152.0	137.1	7.4	30	1	-65
3NPH-D_7-butyrate	229.09	137.1	152.0	2.5	30	1	-30
3NPH-D_4-CDCA[d]	530.33	152.0	137.1	6.8	30	1	-65
3NPH-D_4-DCA[e]	530.33	152.0	137.1	6.2	30	1	-65

[a] UDCA, ursodeoxycholic acid; [b] HDCA, hyodeoxycholic acid; [c] isoDCA, isodeoxycholic acid; [d] CDCA, chenodeoxycholic acid; [e] DCA, deoxycholic acid; [f] 3-DCA, 3-deoxycholic acid.

Table S2. Sample preparation and acquisition time in minutes to acquire quantitative concentration values for one biological sample using conventional metabolomics methods and the rapid metabolomic screen.

	time (min)					
**MS Platform**	**dry down**	**derivatization**	**additional [a]**	**acquisition [b]**	**analysis**	**total**
**QToF LC-MS**	120	0	60	359	10	**549**
**PFB-Br GC-MS**	0	60	20	228	10	**318**
**Rapid Screen**	0	30	10	160	10	**210**

[a] Additional sample preparation for QtoF LC-MS method involves resuspension, centrifugation, and transfer to a new MS vial, additional steps in PFB-Br GC-ECNI-MS involve sample cooling, centrifugation, and transfer to a new MS vial, while additional steps in the rapid metabolomic screen involve sample handling and reaction quenching at -80 °C; [b] Acquisition time includes the blanks, quality control, and calibration curve sample injections required to generate reliable quantification of metabolites in a single biological sample.

Table S3. Summary of performance metrics for rapid metabolite screen.

Performance Characteristic	Butyrate	Deoxycholic acid
**Analytical Measurement Range**	4.30 μM–3030 μM	0.9 μM–64.9 μM
**Lower Limit of Quantification**	3.71 μM	0.7 μM
**Inter-Day Precision** (%CV, n = 14 across 8 runs)	2.43–10.4%	7.4–14.3%
**Recovery** (across 5 concentrations, n = 14)	98.2–109%	92.4–109.7%
**Isomers Accounted For**	isobutyrate	UDCA, HDCA, 3-DCA, CDCA, isoDCA

Table S4. Comparison of butyrate and deoxycholic acid concentration between the rapid metabolomic screen and conventional mass spectrometry-based methods with patient clinical metadata. 'Greater-than' or 'less-than' values indicate metabolite concentrations above and below the AMR for that method.

	Clinical Metadata			Butyrate (μM)		Deoxycholic acid (μM)	
**De-identified Patient Sample ID**	**Patient cohort**	**Race**	**Sex**	**Rapid Screen**	**PFB-Br - GCMS**	**Rapid Screen**	**QToF LCMS**
HT_009_03	Heart transplant	Black or African-American	Male	545.36	< 750	1.82	< 2.50
HT_027_03	Heart transplant	White	Male	2626.80	3460	> 64.90	523.43
HT_032_18	Heart transplant	White	Male	< 4.30	< 750	< 0.90	< 2.50
HT_042_07	Heart transplant	Black or African-American	Female	< 4.30	< 750	< 0.90	< 2.50
HT_048_01	Heart transplant	Black or African-American	Male	145.76	< 750	< 0.90	< 2.50
HT_061_02	Heart transplant	Black or African-American	Male	< 4.30	< 750	< 0.90	< 2.50
HT_066_06	Heart transplant	Black or African-American	Female	57.23	< 750	1.86	< 2.50
HT_071_06	Heart transplant	Asian/Mideast Indian	Female	82.24	< 750	< 0.90	< 2.50
HT_080_04	Heart transplant	Black or African-American	Female	< 4.30	< 750	< 0.90	< 2.50
HT_106_01	Heart transplant	Black or African-American	Male	> 3030.10	3440	> 64.90	1606.54

	Clinical Metadata			Butyrate (µM)		Deoxycholic acid (µM)	
**De-identified Patient Sample ID**	**Patient cohort**	**Race**	**Sex**	**Rapid Screen**	**PFB-Br - GCMS**	**Rapid Screen**	**QToF LCMS**
LD_001_01	Liver disease	Black or African-American	Male	1190.46	1660	8.22	4.87
LD_011_01	Liver disease	White	Female	> 3030.10	3570	30.38	12.07
LD_014_02	Liver disease	White	Male	2866.56	3090	> 64.90	20.62
LD_023_01	Liver disease	More than one Race	Male	2594.94	2860	25.75	8.87
LD_025_03	Liver disease	Unknown or Patient unable to respond	Female	< 4.30	< 750	< 0.90	< 2.50
LD_046_01	Liver disease	Black or African-American	Female	2076.40	2580	15.84	14.90
LD_049_01	Liver disease	White	Male	239.53	840	16.21	14.57
LD_061_01	Liver disease	White	Female	2433.77	2970	35.44	27.49
LD_070_01	Liver disease	Black or African-American	Female	1439.95	1950	5.12	4.25
LD_087_01	Liver disease	Black or African-American	Female	1000.91	920	3.82	4.38
LD_095_01	Liver disease	White	Female	< 4.30	< 750	< 0.90	< 2.50
LD_129_27	Liver disease	Black or African-American	Female	84.56	< 750	< 0.90	< 2.50
LD_134_04	Liver disease	White	Male	< 4.30	< 750	< 0.90	< 2.50
LD_136_08	Liver disease	White	Male	> 3030.10	14960	< 0.90	2.85
LD_155_01	Liver disease	Black or African-American	Male	< 4.30	< 750	< 0.90	< 2.50
LD_156_01	Liver disease	Black or African-American	Male	31.21	< 750	2.68	3.21
LD_159_01	Liver disease	More than one Race	Female	563.70	< 750	3.68	3.19
LD_174_01	Liver disease	Black or African-American	Female	< 4.30	< 750	< 0.90	< 2.50

	Clinical Metadata			Butyrate (µM)		Deoxycholic acid (µM)	
**De-identified Patient Sample ID**	**Patient cohort**	**Race**	**Sex**	**Rapid Screen**	**PFB-Br - GCMS**	**Rapid Screen**	**QToF LCMS**
LD_181_01	Liver disease	Black or African-American	Male	1199.18	1670	4.93	5.66
LD_191_01	Liver disease	More than one Race	Male	2787.93	3430	36.49	38.23
LD_192_01	Liver disease	Black or African-American	Male	> 3030.10	3610	< 0.90	2.53
LD_193_01	Liver disease	White	Female	1908.16	2420	35.35	35.81
LD_194_08	Liver disease	White	Male	> 3030.10	8340	< 0.90	< 2.50
LD_195_01	Liver disease	White	Male	2899.99	3390	61.26	54.81
LD_228_01	Liver disease	Black or African-American	Female	362.53	1010	1.14	< 2.50
LD_234_11	Liver disease	Asian/Mideast Indian	Female	< 4.30	< 750	1.73	< 2.50
LD_242_01	Liver disease	More than one Race	Female	1970.14	2430	5.55	< 2.50
LD_262_01	Liver disease	More than one Race	Male	225.06	810	15.51	7.72
LD_264_06	Liver disease	White	Male	1789.05	2440	> 64.90	143.37
LD_284_02	Liver disease	Patient declines to respond	Female	> 3030.10	4000	< 0.90	< 2.50
LD_293_01	Liver disease	White	Male	2208.65	2560	17.91	7.32
LD_302_01	Liver disease	White	Male	41.27	< 750	< 0.90	< 2.50
LD_304_01	Liver disease	White	Female	2360.53	2430	12.09	7.69
LD_313_01	Liver disease	Black or African-American	Female	27.67	< 750	3.21	< 2.50
LD_314_01	Liver disease	Black or African-American	Female	1458.83	1960	< 0.90	3.36
LD_322_11	Liver disease	White	Female	< 4.30	< 750	< 0.90	< 2.50
LD_338_02	Liver disease	More than one Race	Female	629.40	820	26.35	32.43

	Clinical Metadata			Butyrate (µM)		Deoxycholic acid (µM)	
**De-identified Patient Sample ID**	**Patient cohort**	**Race**	**Sex**	**Rapid Screen**	**PFB-Br - GCMS**	**Rapid Screen**	**QToF LCMS**
LD_347_01	Liver disease	Black or African-American	Female	< 4.30	< 750	< 0.90	2.85
LD_394_01	Liver disease	Black or African-American	Female	2018.87	2350	32.24	43.53
LD_411_02	Liver disease	Black or African-American	Female	281.17	< 750	< 0.90	< 2.50
LD_413_01	Liver disease	White	Male	2263.81	2540	7.03	6.44
LD_414_03	Liver disease	Black or African-American	Male	183.40	< 750	< 0.90	< 2.50
LD_446_01	Liver disease	Unknown or Patient unable to respond	Female	2952.59	3280	> 64.90	62.08
LD_470_01	Liver disease	White	Male	1010.40	1310	8.81	10.44
LD_481_01	Liver disease	White	Female	300.33	< 750	7.69	7.67
LD_494_01	Liver disease	Asian/Mideast Indian	Female	507.24	< 750	10.28	13.69
MICU_063_02	Medical intensive care unit	White	Male	> 3030.10	2350	> 64.90	143.72
MICU_082_03	Medical intensive care unit	Black or African-American	Male	2352.20	2180	1.38	< 2.50
MICU_103_01	Medical intensive care unit	Black or African-American	Female	69.63	250	16.11	2.67
MICU_107_03	Medical intensive care unit	Black or African-American	Female	24.46	< 750	7.72	2.73
MICU_118_06	Medical intensive care unit	White	Male	257.55	< 750	12.92	7.57
MICU_181_01	Medical intensive care unit	Black or African-American	Male	> 3030.10	3860	> 64.90	106.88
MICU_187_01	Medical intensive care unit	Black or African-American	Female	188.92	< 750	> 64.90	58.38
MICU_199_05	Medical intensive care unit	Black or African-American	Male	< 4.30	< 750	17.72	10.34

	Clinical Metadata			Butyrate (μM)		Deoxycholic acid (μM)	
**De-identified Patient Sample ID**	**Patient cohort**	**Race**	**Sex**	**Rapid Screen**	**PFB-Br - GCMS**	**Rapid Screen**	**QToF LCMS**
MICU_205_01	Medical intensive care unit	White	Male	941.73	1110	< 0.90	< 2.50
MICU_238_02	Medical intensive care unit	White	Male	1268.99	< 750	5.59	3.63
MICU_260_03	Medical intensive care unit	White	Male	1742.45	1960	> 64.90	106.78
MICU_318_02	Medical intensive care unit	White	Male	< 4.30	< 750	9.80	8.23
MICU_331_04	Medical intensive care unit	Asian/Mideast Indian	Female	99.90	< 750	14.37	3.92
MICU_367_03	Medical intensive care unit	White	Female	< 4.30	< 750	< 0.90	< 2.50
MICU_426_02	Medical intensive care unit	Black or African-American	Female	2028.07	2600	> 64.90	133.74
MICU_436_04	Medical intensive care unit	Black or African-American	Male	< 4.30	< 750	< 0.90	< 2.50
MICU_494_01	Medical intensive care unit	White	Female	1568.62	1600	< 0.90	< 2.50
UC_021_07	Liver transplant	White	Female	1810.27	1130	> 64.90	278.32
UC_050_02	Liver transplant	White	Female	< 4.30	< 750	< 0.90	< 2.50
UC_077_04	Liver transplant	White	Female	< 4.30	860	< 0.90	< 2.50
UC_136_01	Liver transplant	Black or African-American	Male	2804.84	3820	> 64.90	64.27
UC_149_01	Liver transplant	Black or African-American	Male	> 3030.10	3780	> 64.90	1111.21

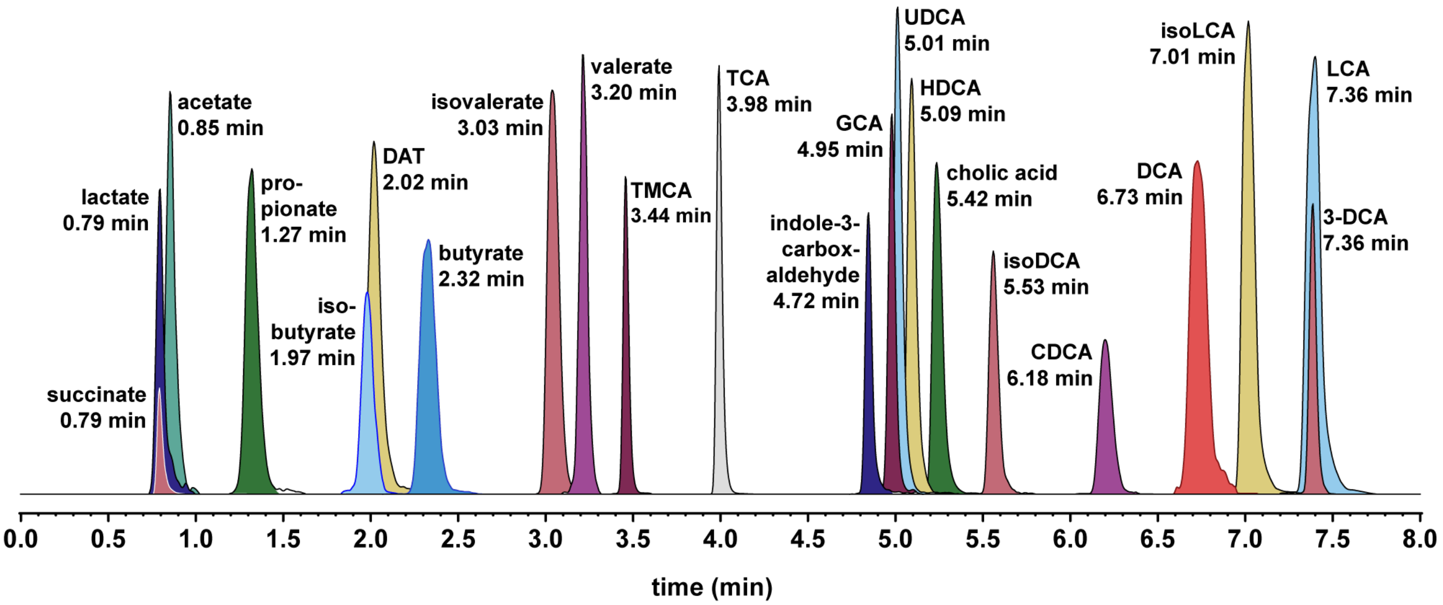

Figure S1. Overlaid extracted ion chromatograms of additional metabolites that can be measured using the rapid screen LC-MS method. DAT, desaminotyrosine; TMCA, tauromuricholic acid; TCA, taurocholic acid; GCA, glycocholic acid; UDCA, ursodeoxycholic acid; HDCA, hyodeoxycholic acid; isoDCA, isodeoxycholic acid; CDCA, chenodeoxycholic acid; DCA, deoxycholic acid; isoLCA, isolithocholic acid; LCA, lithocholic acid; 3-DCA, 3-deoxycholic acid.
